# A collaborative clinical and population-based curriculum for medical students to address primary care needs of the homeless in New York City shelters: Teaching homeless healthcare to medical students

**DOI:** 10.1007/s40037-016-0270-8

**Published:** 2016-06-09

**Authors:** Ramin Asgary, Ramesh Naderi, Margaret Gaughran, Blanca Sckell

**Affiliations:** Department of Medicine, New York University School of Medicine, New York, USA; NYU Lutheran Family Health Centers, New York, NY USA; Independent Consultant, London, UK; Touro College of Osteopathic Medicine, New York, NY USA

**Keywords:** Curriculum, Health disparities, Homeless, Primary care, Medical students

## Abstract

Background Millions of Americans experience homelessness annually. Medical providers do not receive adequate training in primary care of the homeless.

Methods Starting in 2012, a comprehensive curriculum was offered to medical students during their family medicine or ambulatory clerkship, covering clinical, social and advocacy, population-based, and policy aspects. Students were taught to: elicit specific social history, explore health expectations, and assess barriers to healthcare; evaluate clinical conditions specific to the homeless and develop plans for care tailored toward patients’ medical and social needs; collaborate with shelter staff and community organizations to improve disease management and engage in advocacy efforts. A mixed methods design was used to evaluate students’ knowledge, attitudes, and skills including pre- and post-curriculum surveys, debriefing sessions, and observed clinical skills.

Results The mean age of the students (*n* = 30) was 26.5 years; 55 % were female. The overall scores improved significantly in knowledge, attitude, and self-efficacy domains using paired t‑test (*p* < 0.01). Specific skills in evaluating mental health, substance abuse, and risky behaviours improved significantly (*p* < 0.05). In evaluation of communication skills, the majority were rated as having ‘outstanding rapport with patients.’

Conclusions Comprehensive and ongoing clinical component in shelter clinics, complementary teaching, experienced faculty, and working relationship and collaboration with community organizations were key elements.

## What this paper adds

Medical providers often lack the skills to address the unique healthcare needs of the homeless with their social conditions that affect clinical encounters. Structured and formal integrated curricula to specifically address the primary care needs of the homeless, with their specific challenges and barriers, often do not exist. By designing a health disparities clinical and population-based curriculum, we were able to better prepare medical students to address the multi-level barriers to healthcare among the homeless. This piece describes the development, implementation, feasibility, and assessment of efficacy of such curriculum.

## Introduction

Annually, 3.5 million Americans experience homelessness and around 630,000 spend each night in the shelter system [1]. Overwhelmingly, the homeless have lost their housing due to eviction, inability to pay rent, domestic abuse and family disputes [2]. Men aged 45 to 54 years, many of whom are veterans, are at the highest risk [3]. Among this population, age 45 and above, chronic diseases such as heart disease and cancer are consistently the leading causes of mortality, followed by substance abuse [4, 5]. Injuries and other infectious diseases are also common [6]. The rates of smoking and substance abuse are higher than in the general population [6, 7]. The majority of the homeless population belong to racial and ethnic minority groups, and are especially vulnerable to and suffer disproportionately from worse health outcomes than other groups [5].

Multi-level barriers to access to healthcare exist among the homeless, including history of mental illness or substance abuse, fatalistic views about chronic disease, distrust of providers or health systems due to a history of discrimination, and a lack of medical insurance and access to primary care [8–12]. Systems-level barriers include lack of a primary care physician, no clinic visit to a physician in the past year, inadequate provider counselling and subsequent misconceptions about chronic disease management, lack of insurance, and poor access to healthcare in general [8–12]. Providers often have misconceptions, biases or subtle prejudice against the homeless and their medical needs or priorities [12]. Additionally, providers may lack the skills to address the unique social conditions that affect their clinical encounters; they may have difficulty addressing preventive care for this population within the time constraints of the typical medical visit [12]. The focus of the health system has been, at best, on addressing urgent issues of the homeless, while neglecting primary and preventive care [9, 10, 13].

Currently, limited health disparities curricula in medical schools focus on healthcare of the poor or low socioeconomic populations in general, and issues faced by refugees and immigrants [14–19]. Exposure to healthcare of the homeless is often limited or student-driven [20–24]. Structured and formal integrated curricula to specifically address the primary care needs of the homeless, with their specific challenges and barriers, often do not exist. While global health has been emphasized in medical school curricula over the past decade [25–27] domestic opportunities that could improve skills in addressing health disparities (which are likely transferable to other vulnerable populations, nationally and internationally) are often overlooked.

We developed and offered an educational model that originally grew out of a clinical service project introduced by the Community Medicine Program at St. Vincent Hospital in New York City, which was later embraced by the NYU Lutheran Medical Center, to better serve its largely low-income and homeless populations. By designing a health disparities clinical and population-based curriculum, we aimed to better prepare medical students to address the multi-level barriers to healthcare among the homeless, as well as the low-income and poor population. In this piece, we describe the development, implementation, feasibility, and assessment of efficacy of this curriculum.

## Methods

### Curriculum description

The curriculum was developed by two core faculty (RA and BS) and offered in the Community Medicine Program of the NYU Lutheran Medical Center between 2012–2014. Participants were third and/or fourth year medical students who either elected or were assigned to participate in a one-month community medicine rotation to fulfil their primary care or family medicine clinical rotation requirements. The overarching goals were to improve sensitivity toward, and understanding of, the impact of fundamental causes and social determinants on the health of individuals with a low socioeconomic status; awareness regarding healthcare of the homeless and their epidemiology and demographics; understanding the implications and the health consequences of lack of housing, and assessing strategies to address them at the patient and population levels; comfort, efficacy, and effectiveness in developing and implementing a plan of care for a homeless patient; and knowledge of and skills in utilizing available social, community, and governmental resources for such patients. Students were encouraged and supported to explore and discuss policy and advocacy options to improve the health of this frequently ignored population in the interface of social science, public policy, and clinical medicine.

Through a primarily clinical exposure in shelter-based clinics and shelter settings, active faculty precepting, and participating in the primary care model of care at shelter-based clinics, students were expected to achieve specific learning objectives as presented in Tab. 1 [17–21]. The curriculum format included structured reading assignments, weekly lecture series, weekly case presentations, daily team discussion sessions with social workers and staff at shelters and shelter clinics, and clinical sessions with clinical precepting by faculty 3 days a week.

**Tab. 1:** Curriculum objectives for participating medical students; shelter-based clinics, New York City, 2012–4

**Objectives**	**Format/Venues**
To describe epidemiology of homelessness, recognize it as a social problem with health implications, and understand the role of fundamental causes of diseases	Readings Discussion sessions Lectures
To demonstrate skills to investigate and evaluate psychosocial components/stressors of their patients illness	Clinical sessions Clinical precepting
To develop skills to address biomedical problems specific to homeless population including but not limited to consequences of substance abuse, living on streets or in transitions or in shelters	Targeted readings Clinical sessions Clinical precepting
To recognize and address barriers to healthcare access among homeless population (health system level, individual levels, and provider competency level)	Targeted readings Discussion sessions Lectures
To develop skills to efficiently use the primary care setting and its resources to address patient’s socio-medical conditions effectively	Targeted readings Clinical sessions Team discussion
To recognize and apply patient-centred approach considering patient’s priorities	Discussion sessions Clinical precepting Lectures
To develop skills in efficient use of time in primary care setting and apply evidence-based approaches to medical conditions of homeless	Clinical sessions Clinical precepting
To demonstrate skills in working collaboratively with community and grass-root organizations that provide services to homeless and to learn effective team work with case workers, support staff and shelter staff	Team discussion Clinical precepting
To develop skills in recognizing and directing patients to appropriate mental health and substance abuse programmes	Targeted readings Clinical sessions Clinical precepting
To develop skills in efficient use of time in primary care setting and apply evidence-based approaches to medical conditions of homeless	Readings Clinical precepting

The curriculum directors had extensive experience working with underserved populations, including immigrants, refugees, and torture survivors; and curriculum development in medical and public health schools. They also had collaborated with non-governmental organizations in and outside academic settings. Two core faculty (RA and BS) incepted the curriculum by meeting regularly over a period of 2 years to discuss the educational needs of the programme, as well as challenges and resources, both in the clinical and social settings at the shelters; develop relationships with and elicit interest and feedback from social service providers at the shelters and collaborating community organizations; and evaluate opportunities for hands on teaching. The faculty lobbied extensively with both medical school and shelter directors to address logistical issues; elicited feedback from students who elected a rotation at the clinical sites; and researched and reviewed available curricula on disparities to inform the content and format of the curriculum [14, 15, 19–21]. First, a small number of students enrolled in the curriculum to anticipate and address logistical challenges and opportunities before it was open to a larger group. Before each rotation, briefing sessions were held with students in which the logistics and environments of shelters and neighbourhoods, and necessary precautions and processes, were discussed. Students were always paired; occasionally, when there was one student in a shelter, a social worker, support staff, navigator or a faculty member accompanied the student to and out of the shelter or shelter clinic when needed. Students were encouraged to help develop or participate in a research project related to primary care services for the homeless, and participate in future electives.

## Curriculum evaluation

Complementary approaches were used to assess curriculum impact including a) pre- and post-curriculum knowledge, attitude, and self-efficacy surveys, b) formal clinical skills assessments by the faculty preceptor, and c) discussion sessions with students.

To objectively assess students’ performance, anonymous pre- and post-curriculum surveys were administered to students at the beginning and the end of the curriculum, respectively. Surveys covered five main domains: (a) knowledge of regional and national policies and advocacy initiatives regarding the homeless population, (b) knowledge about the demographics and epidemiology of homelessness, and the fundamental social causes of their illnesses, (c) knowledge of the common physical and psychological conditions among the homeless, (d) attitudes and perceptions regarding working with homeless persons, and (e) skills to assess and manage homeless healthcare needs, and advocate on their behalf. The survey questionnaire included multiple-choice questions, selecting one best answer, as well as Likert scale questions regarding attitude and self-efficacy. Data were analyzed using Microsoft Excel 14.0.0, SPSS 20 and QuickCalcs (GraphPad CA, 2014). Parametric or nonparametric statistical tests were performed when appropriate using paired *t-test* measuring mean composite scores within groups (comparing students before and after). Statistical significance was assumed at a *p* < 0.05. Cronbach’s alpha was used as a measure of internal reliability and consistency for questions that were posed on a Likert scale. Electronic or printed curriculum evaluations were used on 22 students in the past 3 years of curriculum implementation.

At the final session of the curriculum, faculty preceptors performed direct clinical skills evaluations that reflected elements (12 items) of history taking, physical exam skills, and communication skills, as well as areas of strengths and weaknesses. All students participated in final face-to-face discussion sessions with the directors to provide feedback. General questions were posed as ice-breakers. Open-ended discussions focused on experienced or perceived challenges, barriers to learning, what and how to improve, and which specific components of the curriculum were more helpful educationally (and why). The majority of these discussions included between 2–4 students over the course of curriculum implementation. Course directors took individual notes, then compared notes, developed coding schemes, reviewed codes and developed themes using a qualitative descriptive approach.

This study received the Institutional Review Board approval from NYU Lutheran Family Health Centers.

## Results

Between 2012 and 2014, 30 medical students participated in the curriculum; however, evaluations were available for 22 students. The eight students participating in the piloting phase of the curriculum did not receive any pre- or post-curriculum survey. Mean age was 26.5 years (SD ± 3); 55 % female; and 65 and 35 % were medical students in their third and fourth year of study, respectively. Ten percent were dual-degree Doctor of Medicine/Master of Public Health students. All but two students were from the Icahn School of Medicine at Mount Sinai, New York, NY. Students showed improvement in knowledge, attitude, and self-efficacy questions. The overall scores in knowledge, attitude, and self-efficacy domains improved significantly, using paired t‑test post curriculum (*p* < 0.01). Specific skills in the evaluation of mental health, substance abuse, and other risky behaviours improved significantly (*p* < 0.05) (Tab. 2 and 3).

**Tab. 2:** Knowledge and attitude among medical students pre- and post-curriculum in New York City shelter clinics, 2012–4

	**Pre** Mean SEM *N*	**Post**	***P*** **value paired** *t-test*
**KNOWLEDGE** **Composite score** (Yes/No, or one correct answer)	0.2822 0.0325 18	0.422 0.022 15	*p* < 0.001
What is the average number of homeless persons who sleep on street each night in New York City? a) 20,000 b) at least 10,000 c) 3–4,000 d) I have no idea	0.17 0.09 18	0.87 0.09 15	*p* = 0.001
What is the percentage of family homelessness among homeless population in the United States? a) 15–25 % b) 30–40 % c) 50–70 % d) I have no idea	0.11 0.08 18	0.33 0.13 15	*p* = 0.082
What is among some of the most common complaints in dropping centres? a) Headache b) abdominal pain c) cough d) feet swelling	0.08 0.08 13	0.62 0.15 13	*p* = 0.015
The highest cost of homeless to society comes from? a) Social services b) food and housing c) outpatient care d) hospital admission due to mental illness	0.79 0.11 14	0.92 0.08 13	*p* = 0.081
What is the ethnicity/race with highest rate of homelessness among chronically homeless in New York City? a) Black b) Hispanic c) Whites d) other e) all are equally at risk	0.53 0.12 17	0.80 0.11 15	*p* = 0.040
**ATTITUDE** **Composite score** ^a^	3.35 0.063 18	3.65 0.056 15	*p* < 0.001
I am comfortable being a primary care provider for a homeless person with major mental illnesses	2.81 0.22 22	3.94 0.11 16	*p* = 0.001
I feel comfortable providing care to different minority and cultural groups	4.10 0.22 22	4.38 0.18 16	*p* = 0.029
I feel generally overwhelmed by the complexity of the problems that homeless people have	3.33 0.17 22	2.56 0.18 16	*p* = 0.003
I enjoy learning about the lives of my homeless patients	3.90 0.17 22	4.63 0.13 16	*p* = 0.003
I generally believe caring for the homeless is not financially viable for my career	2.95 0.18 22	2.56 0.26 16	*p* = 0.096
I feel comfortable to provide care to a homeless person with depression	3.14 0.19 22	4.13 0.09 16	*p* = 0.0001
I feel comfortable to provide care to a homeless person with other mental illnesses	2.90 0.19 22	4.13 0.09 16	*p* = 0.0001
I feel comfortable to provide care to a homeless person with substance abuse	2.81 0.16 22	3.81 0.14 16	*p* = 0.0001
I feel comfortable to provide care to a homeless person with alcohol abuse	2.76 0.15 22	3.94 0.14 16	*p* = 0.0001
I feel comfortable to help uninsured or underinsured persons to better navigate health system	2.33 0.20 22	3.25 0.19 16	*p* = 0.021
I feel comfortable to negotiate plan of care with homeless patients considering their constraints and expectations	3.05 0.18 21	4.00 0.20 16	*p* = 0.006

^a^Likert scale: Strongly Disagree (1) Disagree (2) Neither agree/disagree (3) Agree (4) Strongly Agree (5)

**Tab. 3:** Self-efficacy among medical students pre- and post-curriculum in New York City shelter clinics, 2012–4

SELF-EFFICACY	**Pre** Mean SEM *N*	**Post**	***P*** **value paired** *t-test*
**Composite score** ^a^	3.317 0.067 18	3.695 0.061 12	*p* < 0.001
I believe that I can assess depression in a homeless person	3.33 0.20 21	4.44 0.13 16	*p* = 0.0002
I believe that I can apply Depression score/questionnaire to assess depression in a homeless person	3.62 0.18 21	4.69 0.12 16	*p* = 0.0009
I believe that I can obtain and assess psychosocial issues from a homeless person	3.43 0.15 21	4.25 0.14 16	*p* = 0.0004
I believe that I can assess substance abuse in a homeless person	3.43 0.18 21	4.06 0.19 16	*p* = 0.014
I believe that I can assess alcohol abuse or dependence in a homeless person	3.48 0.16 21	4.19 0.14 16	*p* = 0.002
I believe that I can obtain and assess sexual history from a homeless person	3.95 0.08 21	4.44 0.13 16	*p* = 0.0004
I believe that I can assess smoking history and provide smoking cessation to a homeless person	3.86 0.13 21	4.56 0.13 16	*p* = 0.0003
I believe that I have skills in directing homeless persons to potential psychosocial resources	2.24 0.14 21	3.57 0.22 16	*p* = 0.002
I believe that I have skills in directing homeless persons to potential and accessible biomedical resources	2.24 0.15 21	3.38 0.18 16	*p* = 0.0001
I believe that I can work collaboratively with social service providers and community organizations that provide services to the homeless	3.95 0.18 21	4.38 0.13 16	*p* = 0.047
I believe that I have clinical skills to detect and address most medical problems specific to the homeless population	2.95 0.16 21	4.06 0.11 16	*p* = 0.0001
How has your experience here at Community Medicine Program changed your career choices to go to: Primary care residencies (Emergency Medicine, Internal Medicine, Paediatrics, OBGYN, Preventive Medicine, Family Medicine, General Surgery)	3.11 0.11 9	3.56 0.16 16	*p* = 0.078
How has your experience here at Community Medicine Program changed your career choices to work with the underserved?	3.22 0.22 19	4.13 0.15 16	*p* = 0.011

^a^Likert scale: Strongly disagree (1) Disagree (2) Neither agree/disagree (3) Agree (4) Strongly agree (5)

All students were observed and were precepted during all of their clinical encounters by faculty preceptors, and participated in devising the plans of care for patients and addressing social conditions that impacted their patients’ clinical encounters.

Final evaluations of communication skills by the faculty preceptors were available from 14 students. Eight students were not available for a full evaluation of direct clinical encounter observation by the faculty preceptor due to multiple factors, including scheduling problems due to holidays, sick leave, conflict with medical school exams, and incomplete evaluations. A majority were rated as having ‘outstanding rapport with patients = 3’ on a scale of 1 to 3. History-taking skills assessments included items related to the proper introduction to patients, open-ended questions, characterizing chief complaints, asking along a line of reasoning, adequate and relevant review of social history, and validating patients’ concerns. Most students scored a 3 in all criteria, except 3 students who scored partially complete (2) on some (scale included 1 = incomplete/never, 2 = partially complete/sometimes or 3 = complete/always). All students scored either ‘4 = obtained almost all major and minor details of the case’ or ‘3 = obtained most important information-may have missed some minor details.’ The full physical exam included 4 grades and all students performed either 4 ‘4 = fluid exam and skilfully put patient at ease and included all relevant components’ or ‘3 = performed exam competently, included most important components and was respectful to patients.’ Free text areas included descriptions of individual students’ strengths and weaknesses.

Briefing sessions after the completion of the rotation revealed major themes, including characteristics of clinical exposure, exposure to social determinants of health, collaborative nature of services, and interest in and perceived challenges of working with the underserved. Overwhelmingly, students found the rotation rich in clinical skill learning, exposure to important social factors, and collaborating environment and teamwork at the shelters and shelter clinics. Students reported opportunities to work with mental health professionals, caseworkers, social workers, and patient navigators in shelters and shelter clinics to be very informative and enlightening. Some students elaborated that the experiences provided them with better perspectives on their own personal and professional lives, and served as eye openers. A majority of students indicated that they would seriously consider working with underserved communities in their future careers. The main issues regarding the future career choices of primary care specialties were time constraints and a general lack of system support in most primary care settings. Students in general did not complain about safety or security at shelter or clinic sites. Women’s shelters, where survivors of sexual trauma or abuse resided, were in general reserved for female students, and their working environments were often described as ‘tough’ but ‘manageable’ by the students.

## Discussion

The high number of homeless people in almost every major city in the US [1] provides a valuable opportunity to train practitioners in health disparities, and to address the specific needs of this vulnerable population. Our findings indicate that students gained significant knowledge, attitude, and skills after participating in the clinical and population-based curriculum provided. Despite a high level of baseline attitudes and self-efficacy, there were still significant increases in these educational domains. The curriculum’s unique approach for skills building used a population case-based health disparities focus, highlighting the process by which social determinants of health intersect medical, public health, and social systems and improving comprehensive learning.

### Lessons learned

Overall, the curriculum was a positive experience for the students. They gained an understanding of epidemiology and demographics of the homeless, appreciation for the health consequences of lack of housing, knowledge and skills regarding appropriate social and community resources for the homeless, and skills in addressing them at the individual and population levels. Students gained clinical skills in identification, diagnosis and management of common medical and psychosocial conditions, as well as substance abuse issues and the identification and assessment of the broader socioeconomic and policy factors affecting the healthcare of the homeless.

Students rotated through multiple shelter clinics and were exposed to a wide variety of special shelters, including those specific for significant mental health issues, substance abuse problems, or post incarceration facilities. This provided an opportunity for better development of skills in interviewing and taking care of individuals with more precarious situations, which will ultimately improve their ease and comfort with subsequent homeless patients during their career. For patients, it likely fostered better patient-provider trust and rapport with student providers. To our knowledge and experience, the strength of this curriculum was in its setting and complementary models of teaching clinical and population-based medicine at the intersection of social science, which provided unique clinical and non-clinical learning opportunities. Students appreciated the triangulated teaching approach of structured readings, clinical precepting, and the discussion sessions with medical and social service providers. This created an opportunity for group learning and collaborative teamwork with professionals from other disciplines. The location of our clinics, which were based at the shelters, and the close partnership with community organizations, shelters, and neighbourhood entities, along with a robust social and medical service referral system with the parent hospital, fostered a more effective collaborative environment in which students, faculty, and clinic/shelter staff, caseworkers, and community organizations shared ideas and worked closely to improve the health of each individual homeless patient.

Over the past ten years, there has been a greater interest and movement toward social responsibility in medicine and global health, which includes the appreciation of the effects of social determinants of health, [28, 29] the issues of social justice, the right to healthcare and greater health equity, and the reduction of health disparities [30]. An overwhelming number of homeless people in our shelter clinics are from racial and ethnic minority groups, which helped students improve their cultural competency skills in identifying and addressing the effects of racial and ethnic factors on their patients’ healthcare. This experience has undoubtedly helped our students to better recognize the socioeconomic context of their patients’ illnesses, and to realize vividly the context of health disparities in the United States.

Some important limitations of our curriculum evaluation include the self-reported nature of pre- and post-curriculum surveys, and the possibility of socially desirable responses and feedback. Despite a relatively small number of students, using paired analysis of pre- and post-curriculum responses for each student helped demonstrate statistically significant improvements in educational domains. We used a combination of quantitative and qualitative approaches to better elicit feedback, and faculty preceptors directly evaluated students’ skills at the end of curriculum. While different batches of students participated in the training over the period of curriculum implementation, and have been evaluated by different faculty preceptors, the direct observations and assessments by faculty preceptors may have been prone to grade inflation. The specific setting and context of our experiences and the inner city urban setting in the city of New York, have likely influenced the experience and challenges of our patients, but also the content of the curriculum, experiences of our student participants, and the success of our programme. The majority of the homeless in New York City are from ethnic and racial minority groups, and may face both health system challenges and opportunities that are different from other states, countries, or suburban or rural areas. The demographics of our students, the urban health system setting, and the challenges and availability of social service agencies are also likely to vary in other settings. Our assessment of the curriculum only evaluated its short-term impact, and it was not designed to assess its long-term impact and attainment of educational skills.

### Challenges

We faced common barriers in developing and implementing a customized health disparities curriculum within a primarily service-oriented programme, including a lack of funding, difficulties coordinating and accommodating faculty and trainees’ busy schedules [14, 15], devoting specific didactic weekly sessions on an uninterrupted basis, consistent networking with grass-root and advocacy organizations, and coordinating students’ clinical and didactic sessions with clinical preceptors. Other challenges included securing administrative support from the parent institutions to assure that the training experience and clinical services were uninterrupted. The course directors provided all administrative support and primarily used personal relationships to overcome most of these challenges through close negotiations with the clinics, shelters, and community organizations. No funding was provided to reimburse course directors, collaborating organizations or shelter staff. To our experience, taking the steps to create a formal supervised exposure for medical students during their ambulatory blocks, expert faculty, existing collaborative work with community organizations, and maintaining institutional support during training are essential components. The body of knowledge in regard to health disparities and domestic global health experiences and competencies has significantly improved in recent years. Extensive core faculty experience in population-based medicine, curriculum development, and working with the underserved, immigrants, refugees, and the homeless, along with available literature resources [9, 13–15, 20, 21], helped to set objectives, and to design and implement the curriculum.

## Conclusions

The significant increase in interest among medical students in social responsibility and addressing the social determinants of health has not been matched by adequate preparation and structured clinical and population-based exposures to underserved populations. This relatively novel curriculum, focusing on the healthcare of the homeless, who are overwhelmingly from racial and ethnic minority groups, was designed to teach medical students about the impact of lack of housing and other social determinants on the health of the individual and the population. Considering the sheer number of homeless people, we believe there are opportunities to teach health disparities to medical students in virtually every large city in the United States and abroad. Further emphasis should be placed on incorporating and discussing the interface between clinical medicine, social factors and advocacy opportunities at the population and policy levels in medical schools. Triangulating teaching methods with targeted readings, structured and supervised clinical exposure, and interactive case-based discussion with social service providers; and creating and maintaining a working relationship with service providers, using available community resources and working collaboratively with grass-root organizations, and garnering institutional support and departmental commitment are crucial components.
